# A Comprehensive Review of Modifiable Cardiovascular Risk Factors and Genetic Influences in Dementia Prevention

**DOI:** 10.7759/cureus.48430

**Published:** 2023-11-07

**Authors:** Mariam Khan, Arpita Jaiswal, Bhushan Wandile

**Affiliations:** 1 Medicine, Jawaharlal Nehru Medical College, Datta Meghe Institute of Higher Education and Research, Wardha, IND; 2 Obstetrics and Gynaecology, Jawaharlal Nehru Medical College, Datta Meghe Institute of Higher Education and Research, Wardha, IND

**Keywords:** precision medicine, holistic approach, alzheimer's disease, genetic influences, modifiable risk factors, dementia prevention

## Abstract

Dementia poses a growing global health challenge, demanding a multifaceted approach to prevention. This comprehensive review delves into the interplay between modifiable cardiovascular risk factors and genetic influences in dementia prevention. We examine key risk factors, including hypertension, diabetes, hyperlipidemia, obesity, smoking, and physical inactivity, elucidating their impact on dementia risk and the underlying biological mechanisms. Genetic factors, notably familial Alzheimer's disease (FAD) and the apolipoprotein E (*APOE*) gene, are explored in detail, offering insights into their contributions to dementia susceptibility. Importantly, we highlight the complex interrelationship between genetics and modifiable risk factors, emphasizing the need for personalized prevention strategies. Integrating lifestyle modifications and genetic considerations, a holistic approach is paramount in dementia prevention. Implications for public health initiatives and clinical practices underscore the urgency of tailored interventions. Our call to action urges continued research, precision medicine implementation, and collaborative efforts to mitigate the burden of dementia and enhance cognitive well-being globally.

## Introduction and background

Dementia is one of our most pressing and pervasive global health challenges. As the world's population ages, the prevalence of dementia has surged, imposing immense burdens on individuals, families, and healthcare systems worldwide. The cognitive decline and functional impairment associated with dementia diminish the quality of life for affected individuals and place substantial economic and emotional strains on caregivers and society at large [1-3]. The World Health Organization (WHO) estimates that approximately 50 million people are living with dementia globally, and this number is expected to triple by 2050 if no effective preventive measures are taken. With no cure for most forms of dementia currently available, the imperative to develop strategies for prevention and risk reduction has never been more urgent [4].

Prevention is increasingly recognized as a critical strategy to address the dementia epidemic. While genetics plays a role in some cases, a substantial proportion of dementia cases are attributed to modifiable risk factors, many of which are cardiovascular. This knowledge has opened the door to developing interventions and lifestyle changes that can reduce the incidence of dementia and delay its onset [5]. Moreover, the socioeconomic impact of dementia is staggering, with astronomical healthcare costs and the loss of productive years for those affected and their caregivers. Preventing or delaying the onset of dementia enhances the quality of life for individuals and reduces the economic and social burdens associated with the disease [6].

This comprehensive review explores the interplay between modifiable cardiovascular risk factors and genetic influences in dementia prevention. It will thoroughly examine the key modifiable risk factors associated with cardiovascular health, such as hypertension, diabetes, obesity, and dyslipidemia, and their roles in increasing the risk of dementia. Furthermore, it will explore the impact of lifestyle factors, including physical activity, diet, smoking, and alcohol consumption, on cardiovascular health and dementia risk.

## Review

Modifiable cardiovascular risk factors

Hypertension

Hypertension, or high blood pressure (BP), is a critical factor significantly influencing the risk of developing dementia. Numerous studies have consistently demonstrated a strong link between uncontrolled hypertension and an elevated risk of dementia later in life. This association underscores the importance of addressing hypertension as a preventive measure against cognitive decline [7]. Several mechanisms underlie the connection between hypertension and dementia. Uncontrolled hypertension (including BP value above the autoregulatory limit that maintains constancy in cerebral blood flow hypertension) can adversely affect cerebral blood flow, diminishing the supply of oxygen and essential nutrients to brain cells. This reduced blood flow contributes to cognitive impairment and can lead to dementia. Persistent uncontrolled hypertension can damage the brain's small blood vessels, causing microvascular lesions and white matter alterations closely associated with dementia. Lastly, hypertension may initiate neuroinflammatory processes in the brain, further exacerbating the harm to brain tissue and impairing cognitive function [8].

Fortunately, there are interventions and strategies available for managing hypertension, which can, in turn, help mitigate the risk of dementia. Lifestyle modifications play a pivotal role in hypertension management. Encouraging individuals to adopt a heart-healthy diet, engage in regular physical exercise, maintain a healthy body weight, and practice stress reduction techniques can significantly contribute to blood pressure control. In cases where lifestyle changes are insufficient, prescription medications such as antihypertensive drugs are available and can effectively regulate blood pressure levels. Regular blood pressure monitoring and strict adherence to treatment plans are vital aspects of managing hypertension, as they ensure that blood pressure remains within a healthy range and reduce the associated risk of dementia. In conclusion, the impact of hypertension on dementia risk underscores the importance of preventive measures and timely management to preserve cognitive health and overall well-being [9].

Diabetes

Diabetes, encompassing both type 2 and, to some extent, type 1 diabetes, is a significant factor in increasing the risk of dementia. Poorly managed diabetes can have detrimental effects on cognitive function and accelerate the progression of dementia, emphasizing the importance of addressing diabetes as a risk factor for cognitive decline [10]. Several pathways connect diabetes and dementia. Firstly, insulin resistance and impaired glucose metabolism, which are hallmark features of diabetes, can adversely affect brain function. This can increase the risk of neurodegenerative changes contributing to cognitive impairment. Secondly, diabetes can also lead to vascular problems in the brain, similar to the impact of hypertension. The damage to blood vessels in the brain can hinder proper blood flow and contribute to the risk of dementia. Finally, chronic inflammation is associated with diabetes, and this inflammation may extend its harmful effects to brain tissue, potentially exacerbating cognitive decline [11].

Preventing and controlling diabetes is crucial for overall health and reducing the risk of dementia. Lifestyle modifications play a pivotal role in diabetes prevention and control. Promoting a balanced diet, engaging in regular physical activity, and maintaining a healthy body weight can help regulate blood sugar levels and reduce the risk of developing diabetes. For individuals already diagnosed with diabetes, medications such as insulin and other antidiabetic drugs are available to help manage blood sugar levels effectively. Regular monitoring of blood glucose levels is essential, as it allows for timely adjustments in treatment plans, ensuring that blood sugar remains within a healthy range [12].

Hyperlipidemia

Hyperlipidemia, characterized by elevated levels of low-density lipoproteins (LDLs) and other lipids in the blood, is closely associated with an increased risk of dementia, particularly Alzheimer's disease. Understanding the relationship between elevated LDL cholesterol levels and dementia is crucial for preventive measures and effective management [13]. Biological mechanisms connect hyperlipidemia and dementia. Firstly, elevated LDL cholesterol levels may contribute to the accumulation of amyloid plaques in the brain, a hallmark of Alzheimer's disease. These plaques are thought to disrupt communication between brain cells and lead to cognitive impairment. Secondly, hyperlipidemia can harm the blood vessels in the brain, similar to the effects seen in hypertension and diabetes. This vascular damage can reduce blood flow to critical brain regions, further contributing to cognitive decline [14].

Several therapeutic options are available for managing hyperlipidemia, which can help reduce the risk of dementia. Statins, prescription medications that effectively lower cholesterol levels, are commonly prescribed to individuals with high cholesterol. These drugs work by inhibiting the production of cholesterol in the liver. Additionally, lifestyle modifications are essential in managing hyperlipidemia and reducing the risk of dementia. Encouraging a heart-healthy diet low in saturated fats, engaging in regular physical exercise, and maintaining a healthy body weight are all important steps in managing cholesterol levels. Finally, regularly monitoring cholesterol levels is crucial to assess the effectiveness of treatment and make necessary adjustments to the treatment plan [15].

Obesity

Obesity is a significant contributor to the development of dementia, with a particular association observed with vascular dementia. Excessive body fat can lead to various metabolic disturbances that play a role in cognitive decline, highlighting the importance of addressing obesity as a risk factor for dementia [16]. Several links exist between obesity and dementia. Firstly, obesity triggers chronic inflammation throughout the body, including the brain. This inflammation can harm brain cells and increase the risk of developing dementia. Secondly, obesity often leads to insulin resistance, where cells do not respond well to insulin. This resistance can affect brain function and cognition, potentially contributing to dementia. Lastly, obesity-related vascular problems can impair blood flow in the brain, which is a significant factor in the development of vascular dementia [16].

To prevent and treat obesity and, by extension, reduce the risk of dementia, various strategies are available. Diet and exercise are central to these efforts, as promoting a balanced diet and regular physical activity is crucial for weight management. Encouraging healthy eating habits and promoting physical activity, especially in at-risk populations, are vital for obesity prevention and management. In severe cases of obesity, bariatric surgery may be considered a treatment option to achieve significant weight loss and improve overall health [17].

Smoking

Smoking is a well-established risk factor for dementia, and individuals who smoke are at a higher risk of developing cognitive impairment and Alzheimer's disease. Understanding the association between smoking and dementia is crucial for public health efforts to reduce the incidence of dementia [18]. Several biological pathways connect smoking and dementia. Firstly, smoking generates oxidative stress, producing harmful free radicals that can damage brain cells and DNA. This oxidative damage is thought to contribute to the development and progression of dementia. Secondly, smoking contributes to vascular problems, including narrowing blood vessels and forming plaques, which can impede proper blood flow in the brain. These vascular issues are significant risk factors for various forms of dementia. Lastly, nicotine and other compounds found in tobacco can have direct neurotoxic effects, potentially harming brain tissue and contributing to cognitive decline [19].

To address the risks associated with smoking and reduce the likelihood of dementia, various smoking cessation methods and programs are available. Nicotine replacement therapy (NRT) offers options such as patches, gum, and lozenges to help individuals gradually reduce their nicotine intake while quitting smoking. Additionally, prescription medications such as varenicline and bupropion can aid in smoking cessation by reducing nicotine cravings and withdrawal symptoms. Behavioral support, including counseling and participation in support groups, is also essential for individuals attempting to quit smoking, as it provides the necessary tools and encouragement to overcome the addiction [20].

Physical Inactivity

Physical inactivity significantly contributes to the risk of dementia and cognitive decline. Understanding the relationship between physical inactivity and dementia is vital for public health efforts to preserve cognitive health [21]. Several mechanisms connect physical inactivity and dementia. Firstly, a lack of physical activity can result in reduced blood flow to the brain. This diminished blood flow deprives brain cells of oxygen and essential nutrients, which can contribute to cognitive impairment. Secondly, exercise is crucial in promoting brain plasticity, the brain's ability to adapt and reorganize itself. This plasticity is essential for learning, memory, and overall cognitive health. Thirdly, physical activity improves insulin sensitivity, which has a positive impact on brain function. Insulin resistance, often associated with sedentary lifestyles, can negatively affect brain cells and cognition [22].

To promote physical activity and reduce the risk of dementia, various strategies can be implemented. Public health campaigns can raise awareness about the importance of regular exercise and physical activity for cognitive health. These initiatives can encourage people of all ages to incorporate physical activity into daily routines. Tailored exercise programs designed for different age groups and fitness levels can provide individuals with structured opportunities to engage in physical activity. Additionally, education plays a critical role in promoting awareness of the cognitive benefits of physical activity, motivating people to adopt active lifestyles and prioritizing exercise to preserve cognitive function [23].

Genetic influences in dementia

Overview of Genetic Factors in Dementia

Genetic factors are paramount in the intricate landscape of dementia, exerting a profound influence on the development and progression of this complex neurological condition. These genetic influences provide a window into the intricacies of dementia, shedding light on its origins and risk factors, and they offer invaluable insights that can guide both diagnosis and prevention efforts. Understanding these genetic factors is a fundamental pillar in the study of dementia, as it not only deepens our comprehension of the disease but also has tangible implications for public health and clinical practice [24].

Genetic factors in dementia encompass a spectrum of variations and mutations within an individual's DNA that can either increase or decrease their susceptibility to the disease. The most well-known genetic risk factor for dementia is the apolipoprotein E (*APOE*) gene, with certain variations of this gene, particularly *APOE* *ε4*, strongly associated with an increased risk of Alzheimer's disease. This highlights the importance of genetic testing and counseling in identifying individuals who may be at higher risk and may benefit from early intervention or preventive strategies [25].

Understanding these genetic influences can help identify high-risk individuals and inform preventive strategies. For those with a family history of dementia or specific genetic risk factors, early screening and monitoring become critical tools for identifying potential cognitive changes and initiating interventions at the earliest stages of the disease. Furthermore, genetic research continues to uncover novel insights into the molecular mechanisms underlying dementia, paving the way for innovative therapies and targeted interventions that promise to slow or even prevent the disease [26].

Familial Alzheimer's Disease (FAD)

Familial Alzheimer's disease (FAD) represents a relatively uncommon form of Alzheimer's disease with a direct genetic component, distinguishing it from the more prevalent sporadic form of the condition. The genetics of FAD are characterized by specific mutations in genes associated with amyloid-beta protein production, namely, amyloid protein precursor (*APP*), presenilin-1 (*PSEN1*), and presenilin-2 (*PSEN2*). These mutations are pivotal in the pathogenesis of Alzheimer's disease in affected families. FAD follows an autosomal dominant inheritance pattern, meaning individuals with a single mutated copy of the gene have a 50% chance of passing it on to their offspring [27].

The implications of FAD for dementia prevention and management are multifaceted. Genetic testing for FAD mutations within affected families can be valuable for early diagnosis and targeted interventions. Identifying individuals who carry these mutations enables healthcare providers to closely monitor their cognitive health and initiate preventive measures at an earlier stage. FAD mutations offer unique insights for therapeutic research in Alzheimer's disease. By understanding the genetic mechanisms behind FAD, researchers can develop drug therapies specifically targeting amyloid-beta production. These tailored treatments have the potential to not only benefit individuals with FAD but also inform strategies for managing the more common sporadic forms of Alzheimer's disease, where amyloid-beta accumulation is also a hallmark feature [28].

Apolipoprotein E (APOE) Gene

The apolipoprotein E (*APOE*) gene is a crucial genetic factor that plays a significant role in the risk of developing dementia, primarily Alzheimer's disease. There are three common variants or alleles of the *APOE* gene, *APOE2*, *APOE3*, and *APOE4*, each associated with varying degrees of dementia risk [29].

*APOE4* is a well-established genetic risk factor for late-onset Alzheimer's disease. Carrying one copy of the *APOE4* allele increases the risk of Alzheimer's disease, and having two copies further elevates this risk. Individuals with *APOE4* are more likely to develop Alzheimer's disease than those without this allele. Conversely, the *APOE2* allele is associated with a lower risk of Alzheimer's disease. Individuals with *APOE2* may have a reduced likelihood of developing the condition, providing some level of protection against dementia [30].

To address the risk associated with *APOE*-related dementia, several strategies can be employed. Firstly, lifestyle modification is essential. Individuals carrying the *APOE4* allele can reduce their risk by adopting heart-healthy habits. This includes maintaining a balanced diet low in saturated fats, doing regular physical exercise, managing weight, and controlling other cardiovascular risk factors such as hypertension and hyperlipidemia. These measures can help mitigate the impact of *APOE4* on dementia risk [31].

Cognitive training and mental stimulation are also valuable strategies for individuals with *APOE4*. Engaging in activities that challenge the brain, such as puzzles, reading, or learning new skills, may help maintain cognitive function and delay the onset of dementia [32].

Additionally, regular monitoring and early detection are crucial for individuals with *APOE4*. Periodic cognitive assessments can help detect cognitive changes early, allowing for timely intervention and management. Early detection can also provide an opportunity to implement lifestyle modifications and cognitive training strategies to reduce the impact of *APOE4* on cognitive health [33].

Other Genetic Factors

Dementia is a complex condition with various genetic contributors, in addition to the well-known factors such as familial Alzheimer's disease (FAD) and the *APOE* gene. While FAD and *APOE* are recognized for their significant roles, numerous other genes have been identified that contribute to dementia risk. These genes, often characterized by minor individual effects, collectively form a complex genetic landscape that interacts with other genetic and environmental factors to influence an individual's risk of developing dementia [34].

The implications of these various genetic factors for personalized prevention strategies are noteworthy. Genetic counseling can be a valuable resource for individuals with a family history of dementia. Genetic counselors can assess an individual's specific genetic risk factors, considering their family history and the presence of any known dementia-related genetic variants. This information can help individuals make informed decisions about their cognitive health and potential prevention strategies [35].

Genetic profiling, enabled by advances in genetic research, can play a pivotal role in personalizing prevention strategies. By analyzing an individual's genetic makeup, healthcare professionals can tailor preventive measures to address their specific risk factors. This approach allows for a more targeted and potentially effective approach to dementia prevention, which may include lifestyle modifications, cognitive training, and other interventions [36]. Furthermore, as genetic research advances, the possibility of developing targeted therapies for specific genetic risk factors becomes more promising. While this area of research is still evolving, it holds the potential to revolutionize dementia prevention and treatment by addressing the root genetic causes of the condition [37].

The interplay between modifiable risk factors and genetics

Complex Interactions Between Modifiable Risk Factors and Genetics

The intricate interplay between modifiable cardiovascular risk factors and genetics in dementia prevention represents a multifaceted relationship that defies straightforward categorization. At the heart of this complex interaction is recognizing that genetics can significantly influence how an individual responds to interventions to mitigate these risk factors [38].

Firstly, it is crucial to acknowledge that dementia, including Alzheimer's disease, has a genetic component. Specific genetic variants, such as those associated with the *APOE* gene, are known to increase the risk of developing dementia. However, these genetic factors do not guarantee dementia onset; instead, they interact with various environmental factors, including cardiovascular risk factors, to influence the overall risk profile [39].

In the context of modifiable cardiovascular risk factors such as hypertension, diabetes, hyperlipidemia, obesity, smoking, and physical inactivity, genetics can play a role in determining an individual's susceptibility to these risk factors. Some people may have genetic predispositions that make them more prone to developing these conditions, even without lifestyle-related factors. Conversely, others may have genetic advantages that confer protection against these risk factors [40].

Moreover, genetics can impact how individuals respond to interventions targeting these risk factors. For example, a person with a specific genetic makeup may respond exceptionally well to lifestyle modifications such as diet and exercise, leading to better control of blood pressure, blood sugar, or cholesterol levels. On the flip side, genetic variations may make some individuals less responsive to certain medications commonly prescribed to manage these risk factors [41].

The complexity deepens when considering the cumulative effect of multiple risk factors and their genetic interactions. Some individuals may possess a genetic profile that magnifies the impact of risk factors, while others may have genetic resilience that offers partial protection [42].

Ultimately, the intricate interplay between genetics and modifiable cardiovascular risk factors underscores the personalized nature of dementia prevention and intervention strategies. Genetic testing and personalized medicine approaches hold promise in tailoring preventive measures to an individual's unique genetic makeup. By better understanding these complex interactions, researchers and healthcare providers can develop more effective strategies for mitigating dementia risk and enhancing cognitive health across diverse populations [43].

Examples of how genetics may influence responses to risk factor interventions

Hypertension Management

Genetic variability in drug response: One of the critical factors in hypertension management is recognizing that genetic variability can significantly influence how individuals respond to antihypertensive medications. Pharmacogenomics has shed light on how genetic variations impact drug metabolism, efficacy, and potential side effects. For instance, some individuals may metabolize certain antihypertensive drugs differently, affecting their effectiveness in lowering blood pressure and their tolerance and potential for adverse reactions. By tailoring medication choices based on an individual's genetic profile, healthcare providers can optimize blood pressure control. This personalized approach involves selecting the most effective and well-tolerated medications for each patient, minimizing the need for trial-and-error approaches and enhancing treatment outcomes. Genetic testing can provide valuable insights into which medications will work best for a particular patient, helping to fine-tune hypertension management and improve overall patient care [44].

Genetic predisposition to salt sensitivity: Genetic factors can also play a crucial role in determining an individual's sensitivity to dietary salt, a factor that can significantly exacerbate hypertension. Some people may have a genetic predisposition that makes them more responsive to the adverse effects of high sodium intake. Identifying these individuals through genetic testing can be instrumental in tailoring personalized dietary recommendations as part of hypertension management. Reducing sodium intake is particularly important for those with a genetic predisposition to salt sensitivity. By understanding a patient's genetic predisposition, healthcare providers can create customized dietary plans to reduce salt intake to a level better suited to their genetic makeup. This approach enhances the effectiveness of hypertension management and underscores the importance of precision medicine in addressing individual variations in health and risk factors. Ultimately, genetic insights can empower healthcare professionals to provide more targeted and personalized care for patients with hypertension, potentially improving long-term health outcomes [45].

Diabetes Prevention and Control

Genetic variants affecting glucose metabolism: Genetic variations can profoundly impact how the body processes glucose, a central aspect of diabetes. Different individuals may carry specific genetic profiles that make them more or less prone to insulin resistance, impaired glucose metabolism, or the development of diabetes. By identifying these genetic variants, healthcare professionals and researchers can tailor interventions to accommodate these individual differences. For instance, individuals with a genetic predisposition to insulin resistance might benefit from more intensive lifestyle modifications, closely monitored dietary plans, or specific medications that target their unique metabolic challenges. By customizing interventions based on genetic information, it becomes possible to optimize diabetes prevention and control efforts for each person, potentially preventing the development of the disease or improving blood sugar management [46].

Pharmacogenetics in diabetes treatment: Genetic testing can also be crucial in selecting the most appropriate diabetes medications and dosages for an individual. Pharmacogenetics involves analyzing an individual's genetic makeup to determine how they are likely to respond to specific drugs. For diabetes treatment, genetic testing can help identify the medications that align best with an individual's genetic profile, minimizing the risk of adverse side effects and optimizing treatment outcomes. For example, some individuals may have genetic variations that make them more sensitive to certain diabetes medications, while others may require higher dosages for effective blood sugar control. Pharmacogenetic insights can guide healthcare providers in making informed decisions about medication selection and dosage adjustments, leading to more personalized and effective diabetes management [47].

Hyperlipidemia Management

Genetic basis of cholesterol levels: It is increasingly recognized that genetic factors play a significant role in determining an individual's baseline cholesterol levels. Genetic variations can predispose individuals to high cholesterol levels, even without lifestyle-related risk factors. By understanding these genetic predispositions, healthcare providers can better assess the risk profile of their patients and tailor treatment strategies accordingly. This personalized approach can help determine the intensity of lipid-lowering therapy needed for each individual. For example, individuals with a strong genetic predisposition to high cholesterol may require more aggressive treatments, such as higher statin medications, than those with milder genetic influences. Identifying these genetic factors early on can help prevent cardiovascular complications and may have implications for reducing the risk of dementia in the long term [48].

Personalized statin therapy: Genetic testing has become a valuable tool in managing hyperlipidemia. By analyzing an individual's genetic makeup, healthcare providers can determine the most appropriate statin medication and dosage for that person. Not all statins work the same way for everyone, and some individuals may respond better to specific statins due to their genetic profile. Personalized statin therapy based on genetic testing allows for a more precise and practical approach to managing hyperlipidemia. It can help maximize the benefits of cholesterol reduction while minimizing potential side effects. For instance, a person with a genetic predisposition to a higher risk of statin-related muscle pain may benefit from a different statin or a lower dose to achieve the desired cholesterol-lowering effect without intolerable side effects. This individualized approach enhances the effectiveness of hyperlipidemia management and improves patient compliance and satisfaction with treatment [49].

Obesity Prevention and Treatment

Genetic factors in weight regulation: The role of genetics in weight regulation is well-established, and it is known that genetic variations can significantly impact an individual's propensity to gain weight or respond to specific dietary patterns. These genetic factors can affect various aspects of weight management, including metabolism, appetite regulation, and fat storage. Personalized diet and exercise plans, tailored to an individual's genetic profile, are promising to optimize weight management and improve long-term outcomes. By understanding an individual's genetic predispositions, healthcare providers can design interventions that consider factors such as the individual's ability to metabolize certain nutrients or their likelihood of developing insulin resistance. This personalized approach allows for more effective and sustainable weight loss strategies, as it aligns with the individual's unique genetic makeup [50].

Behavioral genetics: Genetic factors can also influence an individual's susceptibility to cravings, emotional eating, and other behavioral aspects of food choices and consumption. Tailored behavioral interventions, informed by behavioral genetics, can address these genetic predispositions and make it easier for individuals to adhere to healthier lifestyles. For example, individuals with genetic markers associated with a higher risk of emotional eating may benefit from specific cognitive-behavioral strategies to manage stress and emotional triggers for overeating. Additionally, counseling or support groups can provide valuable tools and coping mechanisms for individuals whose genetic makeup may make it more challenging to resist unhealthy food cravings. Recognizing the genetic underpinnings of behavior allows for developing more personalized and practical strategies to combat obesity and promote healthier eating habits [51].

Smoking Cessation

Genetic factors affecting nicotine metabolism: Nicotine, the addictive substance in cigarettes, is processed differently in various individuals due to genetic variations. Some individuals metabolize nicotine rapidly, while others do so more slowly. This variation impacts individuals' response to smoking cessation aids, such as nicotine replacement therapy (NRT). For instance, fast metabolizers may require higher doses of NRT to effectively alleviate withdrawal symptoms, whereas slow metabolizers may achieve the same effect with standard doses. Pharmacogenetics, which explores how genes affect drug responses, enables a personalized approach to smoking cessation. By tailoring interventions based on genetic profiles, healthcare providers can optimize the effectiveness of treatment, increasing the likelihood of successful quitting [52].

Genetic influences on smoking dependence: Genetic influences extend to the level of smoking dependence. Some people are genetically predisposed to become more addicted to nicotine, making it more challenging for them to quit smoking. Understanding an individual's genetic susceptibility to nicotine addiction is crucial in designing effective cessation strategies. Personalized counseling and pharmacotherapy can address these genetic risk factors. For example, individuals with genetic markers associated with heightened dependence may benefit from more intensive counseling or medications specifically tailored to target nicotine addiction. By acknowledging these genetic predispositions, healthcare providers can craft smoking cessation plans that align better with an individual's unique needs, ultimately enhancing their chances of successfully quitting and reducing the risk of relapse [53].

Physical Activity Promotion

Genetic predisposition to exercise response: Genetic predisposition to exercise response is a critical consideration. Genetic variations can influence how a person's body responds to exercise, affecting fitness gains and weight loss. Some individuals may be genetically inclined to experience more substantial improvements in cardiovascular fitness or weight reduction through exercise compared to others. Understanding these genetic differences is key to tailoring exercise plans effectively. By considering an individual's genetic profile, personalized exercise regimens can be crafted to optimize the benefits of physical activity. For instance, if someone possesses genetic markers indicating a high response to aerobic exercise, their exercise plan might emphasize running or swimming to achieve the best results. Conversely, individuals with genetic factors favoring strength training may find resistance exercises more beneficial. These personalized exercise plans enhance the effectiveness of physical activity and make it more motivating, as individuals are more likely to remain engaged and committed when they see noticeable results from their efforts [54].

Genetic determinants of musculoskeletal health: Genetic determinants of musculoskeletal health are another crucial aspect. Genetic factors can increase an individual's susceptibility to musculoskeletal issues during physical activity. This may involve a higher risk of osteoarthritis or tendinopathies, which can impact joint and tendon health when exercising. Recognizing these genetic determinants is vital for guiding exercise recommendations and ensuring that physical activity is safe and tailored to an individual's needs. For example, if someone has a genetic predisposition to joint problems, a fitness program may prioritize low-impact exercises such as swimming or cycling to reduce joint stress. Specific exercises and stretching routines can also be recommended to strengthen and protect vulnerable areas. By considering an individual's genetic factors, healthcare providers and fitness professionals can provide effective exercise guidance that minimizes the risk of injuries and long-term musculoskeletal issues. This personalized approach promotes better physical health and long-term adherence to exercise regimens by making them more suitable and sustainable for each individual's genetic makeup [55].

Importance of Tailored Prevention Approaches

The interplay between genetics and modifiable risk factors underscores the importance of personalized and precision medicine approaches in dementia prevention. Tailored interventions that account for an individual's genetic predispositions, lifestyle, and risk factors can effectively reduce dementia risk. By combining genetic information with targeted lifestyle modifications and medical interventions, healthcare providers can develop comprehensive prevention strategies that address the unique genetic and environmental factors influencing each individual's risk of dementia. Such personalized approaches have the potential to maximize the impact of prevention efforts and improve long-term cognitive health outcomes [56].

Current research and clinical guidelines

Overview of Recent Studies and Findings Related to Dementia Prevention

Lifestyle interventions: Several studies have highlighted the significance of lifestyle modifications in reducing the risk of cognitive decline and dementia. Adopting a heart-healthy diet, engaging in regular physical activity, and participating in cognitive stimulation activities have all been associated with a lower risk of developing dementia. These findings underscore the importance of maintaining a healthy lifestyle to support cognitive health [57].

Blood pressure management: Research has shown that controlling hypertension (high blood pressure) in midlife can significantly impact dementia prevention. Individuals who effectively manage their blood pressure through lifestyle changes and medication have a reduced risk of developing dementia later. This suggests that blood pressure management is crucial to dementia prevention strategies [58].

Diabetes control: Effective management of diabetes, both through medication and lifestyle changes, has been linked to a lower risk of cognitive impairment and dementia. Controlling blood sugar levels can help protect cognitive function, emphasizing the importance of diabetes management in dementia prevention efforts [59].

Genetic research: Ongoing genetic studies have made significant strides in identifying genetic markers associated with dementia risk. These discoveries offer potential targets for future therapeutic interventions and have provided a better understanding of the genetic underpinnings of the disease. While genetics play a role, it is important to note that lifestyle and environmental factors still play a substantial part in dementia risk [60].

Multifactorial approaches: Comprehensive dementia prevention strategies that address multiple risk factors simultaneously have shown promise in reducing the incidence of dementia. These multifactorial approaches recognize that genetics, lifestyle, and medical conditions influence dementia. By targeting various risk factors simultaneously, these interventions aim to provide more effective protection against cognitive decline [61].

Summary of Clinical Guidelines for Dementia Prevention

Cardiovascular health: Managing cardiovascular risk factors is central to dementia prevention. This includes controlling hypertension (high blood pressure), diabetes, and hyperlipidemia (high cholesterol levels). Lifestyle changes should be encouraged, such as adopting a heart-healthy diet and engaging in regular physical activity. Additionally, medications may be necessary for effective management [62].

Smoking cessation: Encouraging individuals to quit smoking is crucial to dementia prevention. Smoking cessation programs and support should be available to help individuals quit this harmful habit, as smoking is a known risk factor for cognitive decline and dementia [63].

Obesity management: Promoting a healthy lifestyle that includes a balanced diet, regular physical activity, and weight control is essential. Obesity is associated with an increased risk of dementia, so maintaining a healthy weight is a key focus in prevention efforts [64].

Cognitive engagement: Staying mentally active and engaged is vital for brain health. Activities such as solving puzzles, reading, learning new skills, and participating in cognitive exercises can help maintain cognitive function and reduce the risk of dementia [65].

Social engagement: Maintaining social connections and staying socially active is another essential recommendation. Social engagement has been linked to better cognitive health, and it can help combat feelings of isolation and loneliness, which are associated with dementia risk [66].

Genetic counseling: For individuals with a family history of dementia or known genetic risk factors, genetic counseling and testing should be considered. Understanding one's genetic predisposition to dementia can provide valuable information for personalized prevention strategies and early intervention [67].

Individualized approaches: Dementia prevention should be tailored to each individual's risk factors and genetic profile. Recognizing that risk factors and genetic influences vary widely, healthcare providers should customize prevention strategies to suit each person's unique needs and circumstances [68].

Gaps in Current Knowledge and Areas for Further Research

Long-term studies: Conducting more extensive and long-term studies is crucial to comprehensively assess the cumulative effects of risk factor management and lifestyle modifications on dementia risk. This can help determine interventions' optimal duration and intensity and their impact on long-term cognitive health [69].

Precision medicine: The field of dementia prevention would benefit from a more personalized approach that considers individual genetic and environmental factors. Research should focus on developing precision medicine strategies that tailor prevention efforts to an individual's unique genetic makeup, lifestyle, and health history [70].

Intervention optimization: To maximize the effectiveness of interventions for dementia prevention, further research is needed to refine and optimize drug therapies, lifestyle interventions, and other preventive measures. This includes identifying the most appropriate timing and dosages for interventions and understanding how they interact with each other [71].

Understudied risk factors: While some risk factors for dementia, such as hypertension and diabetes, have been extensively studied, less attention has been paid to factors such as sleep quality, exposure to environmental toxins, and dietary patterns. Investigating these understudied risk factors can provide valuable insights into additional ways to mitigate dementia risk [72].

Health disparities: Research should explore how socioeconomic factors, access to healthcare, and health disparities impact dementia risk and prevention. Understanding how these factors affect different populations can inform strategies to reduce health inequities related to dementia [73].

Biomarkers: Developing reliable biomarkers for early detection and monitoring of dementia risk and progression is critical. Biomarkers can aid in identifying individuals at risk before cognitive decline becomes severe, allowing for early intervention and personalized care [74].

Global approaches: Extending dementia prevention research to diverse populations and regions is essential. Cultural and environmental variations can influence dementia risk and prevention strategies. Global research efforts can provide a more comprehensive understanding of the disease and its prevention on a global scale [2].

## Conclusions

In conclusion, the comprehensive review of modifiable cardiovascular risk factors and genetic influences in dementia prevention underscores the intricate nature of this multifaceted challenge. Our examination has illuminated the pivotal roles that hypertension, diabetes, hyperlipidemia, obesity, smoking, and physical inactivity play in dementia risk while also emphasizing the significant genetic factors, such as familial Alzheimer's disease and the *APOE* gene, that contribute to an individual's susceptibility. Moreover, the interplay between these genetic influences and modifiable risk factors has highlighted the necessity of personalized prevention strategies. As we progress, it is abundantly clear that a holistic approach, combining lifestyle modifications with genetic considerations, is key to effective dementia prevention. The implications for public health and clinical practice are profound, calling for heightened awareness, precision medicine, and tailored interventions. Our call to action is to continue advancing research, prioritize public health initiatives, and integrate personalized prevention approaches to address this global health challenge and pave the way for a future with reduced dementia prevalence and improved cognitive well-being.
